# Exploring Blood Lead Level Determinants in Refinery Workers: A Cross-Sectional Study

**DOI:** 10.7759/cureus.63330

**Published:** 2024-06-27

**Authors:** Luay M Mohammed, Manoochehr Karami, Yadollah Mehrabi, Seyed S Hashemi, Somayeh Farhang Dehghan, Mohammed Rafiee, Hasan Baiee

**Affiliations:** 1 Department of Epidemiology, School of Public Health and Safety, Shahid Beheshti University of Medical Sciences, Tehran, IRN; 2 Environmental and Occupational Hazards Control Research Center, Research Institute for Health Sciences and Environment, Shahid Beheshti University of Medical Sciences, Tehran, IRN; 3 Department of Environmental Health Engineering, School of Public Health and Safety, Shahid Beheshti University of Medical Sciences, Tehran, IRN; 4 Department of Public Health and Community Medicine, Hilla University College, Babylon, IRQ

**Keywords:** cross-sectional study, iraq, kirkuk, refinery workers, elevated blood lead levels, lead exposure

## Abstract

Background: Occupational lead exposure poses a significant risk to workers in industrial settings, especially in petroleum refineries. The study aimed to examine the prevalence and determinants of high blood lead concentrations among refinery workers in Kirkuk, Iraq. It has also been aimed to provide evidence-based approaches to identify associated risk factors.

Methods: A cross-sectional study was conducted among 187 workers from three departments (transportation, storage, and production) in a petroleum refinery from August 2023 to April 2024. Blood lead levels (BLLs) were measured using graphite furnace atomic absorption spectroscopy (GFAAS) (Agilent, Santa Clara, CA). The elevated BLLs were defined as lead levels in blood samples greater than 10 µg/dL. Data on demographic characteristics, such as occupations, smoking habits, and drinking milk, were collected using a researcher-developed information sheet. Statistical analyses included the Kruskal-Wallis test and Pearson Chi-Square test, and logistic regression was used to address the determinants of elevated BLLs. The corresponding associations were reported using odds ratios (OR) and adjusted OR (AOR) along with 95% confidence intervals (CIs).

Results: Eighty-five percent of the workers had high BLLs, with a median BLL of 17.11 µg/dL. The findings revealed that workers employed in outdoor settings had 4.25 times higher AOR (95% CI: 1.24-14.48) of experiencing high BLLs compared to those working indoors, after adjusting for other factors. This was especially true for workers who spent nine to 16 hours outdoors. Additionally, age and smoking were also found to be associated with an increased risk of high BLLs. On the other hand, the analysis indicated that drinking milk had a protective effect against high BLLs.

Conclusion: The high prevalence of elevated BLLs among refinery workers in Kirkuk underscores the urgency for immediate interventions. Regular monitoring of BLLs, improved training, dietary adjustments (e.g., consuming calcium and phosphate-rich milk, which can help lower BLLs), and smoking cessation programs are recommended to reduce lead exposure and safeguard workers' health. Furthermore, the results suggest that drinking milk could potentially reduce BLLs among petroleum refinery workers. Additional research is necessary to evaluate the effectiveness of these interventions and to continue monitoring exposure levels.

## Introduction

Occupational exposure to lead is a well-recognized health hazard, particularly in industrial settings such as petroleum refineries. Lead, a heavy metal, can cause a range of adverse health effects, including neurological, cardiovascular, renal, and reproductive disorders [1]​​. The toxic effects of lead are dose-dependent, and even low levels of exposure can have significant health impacts, particularly on the nervous system [2].

In petroleum refineries, workers are potentially exposed to lead through various sources, including leaded gasoline residues, and industrial processes [3]. Despite advancements in occupational health and safety standards, lead exposure remains a significant concern in many developing countries due to lax regulations and insufficient enforcement [4]. This is particularly relevant in Iraq, where industrial health and safety practices are often compromised due to ongoing socio-economic challenges [5].

Lead exposure occurs through multiple routes, including inhalation of lead-containing dust or fumes, ingestion of lead-contaminated food or water, and dermal contact with lead-containing materials [6]. In occupational settings, factors, such as the duration of employment and working conditions, play a critical role in determining lead exposure levels. Longer durations of employment in environments with lead exposure correlate with higher cumulative exposures and, consequently, higher blood lead levels (BLLs) in workers [7].

Outdoor workers may face higher risks of lead exposure due to airborne lead particles and potential contact with contaminated soil, which are more prevalent in outdoor industrial environments [8]. Studies have shown that lead in the air and soil is more common in outdoor industrial settings than in other workplaces [9]. This increased environmental lead contamination can lead to higher exposures and consequently, elevated BLLs in outdoor workers who come into contact with these lead-containing materials [10].

Furthermore, nutritional deficiencies in essential minerals, such as iron, calcium, and zinc, can increase the body's absorption of lead. As these deficiencies can exacerbate the effects of lead exposure, they can lead to higher BLLs among workers who have direct contact with lead in their work environment [11].

Kirkuk, a city located in northern Iraq (300 km from the capital, Baghdad) with a notable presence of petroleum refineries, represents a critical area for assessing occupational health risks associated with lead exposure. Previous studies have highlighted the elevated BLLs among industrial workers in similar settings, underscoring the need for systematic evaluation and intervention [12,13]. However, there was a lack of research specifically focusing on the issue of occupational lead exposure, high BLLs, and associated risk factors among petroleum refinery workers in Kirkuk, Iraq.

The study has aimed to fill this gap by assessing the BLLs among workers in the petroleum refineries of Kirkuk, Iraq. A cross-sectional design was employed to measure BLLs among workers in the refinery sites. Additionally, the practices related to lead exposure among the workers were evaluated. By identifying the extent and determinants of elevated BLL, this study seeks to inform targeted interventions and policy measures to protect the health and well-being of refinery workers in Kirkuk.

## Materials and methods

Study design and setting

This cross-sectional study was conducted among workers at petroleum refineries in Kirkuk, Iraq, from August 2023 to April 2024. The primary objective was to evaluate the levels of blood lead and identify associated risk factors among these workers.

Study participants

We recruited 187 workers from three different departments within the petroleum refineries: the transportation department (n=56), the storage department (n=65), and the production department (n=66). Inclusion criteria were workers who had been employed at the refinery and provided informed consent to participate. Exclusion criteria included workers with pre-existing health conditions that could independently influence BLLs, such as renal disease.

Sampling Methodology

The study utilized a stratified random sampling approach to ensure adequate representation of specific worker groups through observation. The workers were categorized into three distinct strata based on their job roles: production department workers with direct contact with petrol, transportation department workers with intermittent contact with petrol, and storage department employees with little direct contact with petrol. This stratification was chosen because the level of lead exposure was expected to vary significantly among these different employee groups.

Sample Size Determination

The sample sizes for the respective strata were determined proportionally based on the total number of individuals in each group - production department workers (50%), transportation department workers (30%), and storage department personnel (20%). This proportional representation ensured that the study sample accurately reflected the distribution of these subgroups within the broader population.

Implementation of Random Sampling

Within each distinct stratum, participants were randomly selected using a computer-generated random number table. This process ensured that each individual within a given stratum had an equal probability of being selected for the study, thereby minimizing potential biases.

Data collection

The researchers gathered information on demographic characteristics, occupations, smoking habits, and dietary practices through a researcher-developed information sheet with closed-ended questions and conducted face-to-face interviews to obtain the data. The independent variables included age (categorized as >40 years and ≤40 years), education level (secondary, institute diploma, bachelor's), and duration of employment (1-10 years, 11-20 years, ≥21 years). Specific employment duration was used to capture potential differences in cumulative lead exposure and its impact on BLLs among the workers while maintaining a manageable sample size for each group. The workers were selected from the transportation, storage, and production departments based on their work role and contact with petrol or additives. The “working outdoors” variable was categorized by whether participants worked outdoors (yes/no) and the hours of outdoor work per day (0-8 hours, 9-18 hours), enabling a detailed assessment of exposure levels. Smoking was categorized by whether the worker smoked (yes/no) and the amount smoked per day (one packet or less, two packets or more), while the “drinking milk” variable was categorized by whether the worker drank milk before coming to work (yes/no) and the number of cups consumed per day (one cup or less, two cups) to assess whether milk consumption was associated with BLLs among the refinery workers. This categorical approach aligned with the data collected through the attached information sheet (please see Supplemental information).

Validity and reliability of the data collection instrument

Validity

To ensure the validity of the data collection instrument, several steps were undertaken.

Content validity: The information sheet was reviewed by a panel of five experts in occupational health and public health science. Their feedback was incorporated to ensure that the items were relevant and comprehensive, thoroughly covering all aspects of lead exposure and its potential health effects.

Construction validity: An exploratory factor analysis was conducted to assess the construct validity of the information sheet. The results indicated that the items loaded appropriately onto the expected factors, supporting the theoretical structure of the instrument.

Criterion validity: Criterion validity was established by comparing information sheet scores with those from previously validated instruments measuring similar constructs. Strong correlations were found, supporting the criterion validity of the instrument.

Reliability

To ensure the reliability of the data collection instrument, the following steps were taken.

Internal consistency: Cronbach's alpha was calculated for the information sheet, yielding a value of 0.85, indicating high internal consistency among the items.

Test-retest reliability: Test-retest reliability was assessed by administering the information sheet to a subset of participants (n=30) on two separate occasions, two weeks apart. The correlation between the two sets of scores was 0.78, indicating good stability over time.

Inter-rater reliability: Inter-rater reliability was assessed for sections involving subjective judgments by having three independent raters evaluate the same responses. Cohen’s kappa was calculated to be 0.72, indicating substantial agreement among raters.

BLL measurement

Blood samples were collected from each participant to measure BLLs. The medical technician put on gloves, washed their hands with liquid soap, and wore personal protective equipment while taking blood. Before drawing blood, the arm was swabbed with alcohol. Venous blood samples (3 mL) were drawn into lead-free EDTA tubes to avoid contamination. The samples were immediately stored at 4°C and transported to the laboratory for analysis within 24 hours. BLLs were determined using graphite furnace atomic absorption spectroscopy (GFAAS) (Agilent, Santa Clara, CA), following the protocol recommended by the Centers for Disease Control and Prevention (CDC) [14]. Quality control procedures included the use of standard reference materials and participation in proficiency testing programs to ensure accuracy and precision. BLL was considered the dependent variable and the main outcome, we defined the elevated BLL as high BLL and categorized the level of lead in blood samples into two categories high (>10 µg/dL) and low (≤10 µg/dL) levels [15].

Statistical analysis

Data were analyzed using SPSS version 26 (IBM Corp., Armonk, NY). Descriptive statistics, including means, medians, and standard deviations, were used to summarize the data. The Kruskal-Wallis test was used to compare BLLs across different departments due to the non-normal distribution of BLL data. The Pearson Chi-Square test was employed to examine associations between categorical variables and BLL categories. The determinants of elevated BLLs have been addressed using logistic regression, with the corresponding associations reported using odds ratios (ORs) along with 95% confidence intervals (CIs). A forward stepwise (likelihood ratio) method was used to select significant predictors for the multivariable model. Cronbach's alpha and Cohen's kappa were calculated to assess the reliability of the data collection instrument [16]. A P-value of <0.05 was considered statistically significant.

Ethical considerations

All participants provided written informed consent prior to their inclusion in the study. The consent process involved explaining the study's purpose, procedures, potential risks, and benefits to the participants in their native language to ensure full understanding. Participants were assured of their right to withdraw from the study at any time without any consequences.

## Results

The findings in Table 1 have presented the median BLLs of the workers in the different departments of the petroleum refinery. The results showed that the median BLL among the 66 workers in the production department was 17.65 µg/dL. The median BLL was 15.65 µg/dL among the 65 workers in the storage department and 17.40 µg/dL among the 56 workers in the transportation department. Overall, the production department had higher median BLLs compared to the other departments.

**Table 1:** Comparison of median and interquartile range of blood lead levels between different departments of petroleum refinery workers *P-value obtained from the Kruskal-Wallis test ^Q1 is quantile one and, Q3 is quantile three

Variable	Departments	n	Blood lead level (µg/dL) (n=187)			
			Median (Q1, Q3^)	Minimum	Maximum	P-value
Petroleum refinery workers	Transportation department	56	17.40 (12.40, 22.01)	7.50	32.30	0.037 *
	Storage department	65	15.97 (10.60, 21.36)	6.65	31.39	
	Production department	66	17.65 (13.22, 22.38)	4.89	43.35	
Total		187	17.11 (12.00, 21.72)	4.89	43.35	

The findings presented in Table 2 showed the distribution of BLLs among the 187 study participants. Out of the total sample, 159 workers (85.0%) had elevated BLL (>10 µg/dL), while 28 (15.0%) had lower BLL (≤10 µg/dL). The participants had a mean age of 41.82±9.60 years.

**Table 2:** Distribution of blood lead level among study participants (n=187) * P-value of Pearson Chi-Square

Variables	Elevated BLL (>10µg/dL)	Lower BLL (<=10 µg/dL)	Total	P-value*	Chi-Square value
	n(%)	n(%)			
Total sample	159 (85.0)	28 (15.0)	187		
Age group(years) (mean±SD)	41.82±9.6				
<=40	34 (65.4)	18 (34.6)	52	0.001	21.828
>40	125 (92.6)	10 (7.4)	135		
Education levels					
Secondary	62 (96.9)	2 (3.1)	64	0.001	14.313
Institute Diploma	55 (84.6)	10 (15.4)	65		
Bachelor's	42 (72.4)	16 (27.6)	58		
Duration of employment (years)					
1 – 10	9 (52.9)	8 (47.1)	17	0.001	17.861
11 – 20	80 (84.2)	15 (15.8)	95		
>=21	70 (93.3)	5 (6.7)	75		
Working outdoor					
No	18 (62.1)	11 (37.9)	29	0.001	14.209
Yes	141 (89.2)	17 (10.8)	158		
Hours of outdoor work/day					
0-8	58 (82.9)	12 (17.1)	70	0.003	8.558
9-18	85 (96.6)	3 (3.4)	88		
Smoking					
No	74 (75.5)	24 (24.5)	98	0.001	14.647
Yes	85 (95.5)	4 (4.5)	89		
Amount smoked daily					
1 packet or less	45 (95.7)	2 (4.3)	47	0.908	0.013
2 packets or more	40 (95.2)	2 (4.8)	42		
Drinking milk					
No	99 (90.0)	11 (10.0)	110	0.023	5.190
Yes	60 (77.9)	17 (22.1)	77		
Number of cups of milk drank					
1 cup or less	34 (79.1)	9 (20.9)	43	0.785	0.075
2 cups	26 (76.5)	8 (23.5)	34		

The study found significant associations between elevated BLL and several factors among petroleum refinery workers, including age, education level, duration of employment, working outdoors, hours of outdoor work per day, smoking, and drinking milk.

The data indicate that older workers, aged 40 years and above, exhibited a higher likelihood of elevated BLL, with 92.6% of this age group demonstrating elevated BLL, compared to their younger counterparts. Furthermore, the findings suggest an inverse relationship between educational attainment and BLL, where workers with lower levels of education, such as those with only secondary education, exhibited a higher prevalence of elevated BLL (96.9%) compared to those with higher educational levels.

Workers with longer employment durations were associated with higher BLL levels, especially for those who had worked 21 years or more (93.3%) compared to workers with shorter employment duration. Additionally, workers who worked outdoors and those who spent more hours working outdoors were significantly more likely to have higher BLL, with 89.2% of outdoor workers exhibiting elevated levels.

Workers who smoked were associated with elevated BLL, whereas workers who drank milk seemed protective, with workers who did not drink milk more likely to have higher BLLs (90%). However, the amount smoked daily and the number of cups of milk consumed daily by workers did not show a significant association with BLLs among refinery workers. These findings highlight the critical demographic and lifestyle factors influencing BLLs in the study population.

The data presented in Table 3 showed the OR and adjusted OR (AOR) for elevated BLLs (BLL) (>10 µg/dL) among 187 workers in petroleum refineries, considering various factors. Workers with longer employment durations (more than 21 years) had significantly higher odds of elevated BLL compared to workers with lower work durations in the refinery (OR = 12.44, p < 0.001). Similarly, those working outdoors had significantly higher odds of elevated BLL compared to indoor workers (OR = 5.06, p < 0.001; AOR = 4.25, p = 0.021).

**Table 3:** Associations between general characteristics and elevated blood lead levels among study participants ª Odds ratio crude from binary logistic regression ᵇ Adjusted odds ratio according to Forward Stepwise (Likelihood Ratio)

Variables	n	Elevated Blood lead level (>10µg/dL) (n=187)			
		OR (95% CI) ^a^	P-value	AOR (95% CI) ^b^	P-value
Duration of employment (years)					
1 – 10	17	Ref.			
11 – 20	95	4.74 (1.57-14.24)	0.006	-	-
>=21	75	12.44 (3.34-46.36)	<0.001	-	-
Working outdoor	158	5.06 (2.06-12.51)	<0.001	4.25 (1.24-14.48)	0.021
Hours of outdoor work/day					
0-8	70	Ref.			
9-18	88	1.70 (0.53-5.51)	0.375	-	-
Age group (years)					
<=40	52	Ref.		Ref.	
>40	135	6.62 (2.79-15.65)	0.002	5.74 (1.88-17.48)	0.004
Education levels					
Secondary	64	Ref.			
Institute Diploma	65	0.17 (0.04-0.84)	0.030	-	-
Bachelor's	58	0.08 (0.02-0.34)	0.001	-	-
Smoking	89	6.89 (2.29-20.77)	0.001	5.43 (1.46-20.12)	0.006
Amount smoked daily					
1 packet or less	47	Ref.			
2 packets or more	42	1.13 (0.15-8.36)	0.908	-	-
Drinking milk	77	0.39 (0.17-0.89)	0.026	0.23 (0.08-0.74)	0.005
Number of cups of milk drank					
1 cup or less	43	Ref.			
2 cups	34	0.86 (0.29-2.53)	0.785	-	-

Older workers (over 40 years of age) in the refinery also exhibited higher odds of elevated BLL compared to younger workers (OR = 6.62, p = 0.002; AOR = 5.74, p = 0.004). Additionally, smokers had significantly higher odds of elevated BLL compared to those who were non-smokers (OR = 6.89, p = 0.001; AOR = 5.43, p = 0.006).

Conversely, the workers who had higher education levels were associated with significantly lower odds of elevated BLL (Bachelor's degree: OR = 0.08, p = 0.001) compared to workers who had lower education levels. The workers who were drinking milk were also found to be protective against elevated BLL (OR = 0.39, p = 0.026; AOR = 0.23, p = 0.005).

However, the number of hours of outdoor work per day and the amount smoked daily did not show significant associations with elevated BLL among refinery workers. These findings emphasize the impact of occupational and lifestyle factors on BLLs among workers in petroleum refineries.

## Discussion

This discussion will compare the results with existing literature, interpret the implications, and suggest potential interventions. Our study revealed that 85.0% of the workers had elevated BLLs (>10 µg/dL), a threshold identified by the CDC as indicative of significant lead exposure [15]. This high prevalence of elevated BLLs is consistent with findings from similar industrial settings, where workers are routinely exposed to lead through occupational activities [12,17]​​. The median BLL of 17.11 µg/dL observed in this study is significantly elevated compared to the CDC's threshold for lead exposure, highlighting the need for urgent intervention.

The study identified significant differences in BLLs across different departments, with the highest levels observed in the production department. This is likely due to direct involvement in processes that emit lead particles and fumes, such as refining and handling of lead materials [18]​​. Workers in the transportation and storage departments also exhibited elevated BLLs, though to a lesser extent, likely due to indirect exposure through contaminated dust and equipment.

Our findings are consistent with earlier studies, which have similarly demonstrated a positive association between BLLs and duration of employment [13]. In the present investigation, the duration of employment was strongly correlated with elevated BLLs, with workers employed for 21 years or more exhibiting significantly higher levels compared to those with shorter tenures. This observed relationship reflects the effect of cumulative lead exposure over time, underscoring the chronic and progressive nature of lead accumulation in the body.

Both age and longer exposure durations can lead to physiological changes that affect lead metabolism and retention in the body [19]. Consistent with these findings, our results showed that older workers (>40 years) had higher BLLs. This could be attributed to the cumulative effects of prolonged lead exposure over the course of their careers. The identification and management of health problems related to lead exposure, particularly in older individuals who are more vulnerable, require age-specific interventions and regular health screenings to address the increased susceptibility observed in this older worker population.

Smoking was significantly associated with higher BLLs, corroborating findings from other studies that have demonstrated enhanced lead absorption in smokers due to inhalation of lead-contaminated smoke particles [20]. Interestingly, milk consumption was found to be protective against elevated BLLs. Calcium in milk competes with lead for absorption in the gastrointestinal tract, thus reducing lead uptake [21]​​. This suggests that dietary interventions could be a viable strategy to mitigate lead absorption among exposed populations.

Educational level emerged as a significant factor in determining BLLs. Workers with higher education levels (bachelor's degree or higher) were less likely to have elevated BLLs compared to those with lower educational attainment. This is consistent with studies suggesting that higher education levels correlate with better knowledge and practices regarding occupational health and safety [22]. Improved education and training programs focused on lead exposure risks and safe work practices are crucial for reducing occupational lead exposure.

Further research

In the future, further research should continue to monitor lead exposure and evaluate the effectiveness of implemented interventions to ensure the ongoing protection of workers' health. The focus should be on longitudinal assessments to better understand causal relationships and evaluate the effectiveness of intervention programs.

Limitations

While this study offers valuable insights into the determinants of elevated BLLs, it is important to acknowledge several limitations. The cross-sectional design of the study precludes causal inference. Furthermore, it is not possible to discriminate the effect of lead from the petroleum industry source from that of other environmental sources. However, all groups in our study have similar exposure to lead.

## Conclusions

This study provides a comprehensive assessment of occupational lead exposure among workers in petroleum refineries in Kirkuk, Iraq. The findings indicate a high prevalence of elevated BLLs among workers, with significant variations observed based on department, duration of employment, working outdoors, age, smoking habits, and dietary practices. Addressing occupational lead exposure requires a multifaceted approach that combines policy interventions, workplace practices, and individual lifestyle changes. By prioritizing these measures, it is possible to create a safer and healthier work environment for petroleum refinery workers in Kirkuk and similar industrial settings. The researchers recommend a multifaceted approach, including implementing routine blood lead screening programs, providing training and education on lead hazards and safety, promoting dietary changes to mitigate lead absorption, establishing comprehensive health monitoring programs, and integrating smoking cessation support for affected workers. These targeted interventions are necessary to effectively protect the health and well-being of refinery workers.

## Appendices

**Table 4:** Information sheet

Demographical characteristics		
Question	Options checked	Score/unit/codes of answer
What department do you work in at the refinery?	Transportation department Storage department Production department	1 2 3
Age	Years	Continues
Education levels	Secondary Institute Diploma Bachelor's	0 1 2
Lifestyle characteristics		
Duration of employment	Years	Continues
Working outdoors?	Yes No	1 0
If yes:		
How many hours of outdoor work/day?	0-8 hours 9-18 hours	0 1
Do you smoke?	Yes No	1 0
If yes:		
How much smoke per day?	1 packet. or less 2 packets or more	0 1
Do you drink milk before coming to work?	Yes No	1 0
If yes:		
How many cups of milk do you drink?	1 cup or less 2 cups	1 0
